# Chinese experts’ consensus on the application of intensive care big data

**DOI:** 10.3389/fmed.2023.1174429

**Published:** 2024-01-08

**Authors:** Longxiang Su, Shengjun Liu, Yun Long, Chaodong Chen, Kai Chen, Ming Chen, Yaolong Chen, Yisong Cheng, Yating Cui, Qi Ding, Renyu Ding, Meili Duan, Tao Gao, Xiaohua Gu, Hongli He, Jiawei He, Bo Hu, Chang Hu, Rui Huang, Xiaobo Huang, Huizhen Jiang, Jing Jiang, Yunping Lan, Jun Li, Linfeng Li, Lu Li, Wenxiong Li, Yongzai Li, Jin Lin, Xufei Luo, Feng Lyu, Zhi Mao, He Miao, Xiaopu Shang, Xiuling Shang, You Shang, Yuwen Shen, Yinghuan Shi, Qihang Sun, Weijun Sun, Zhiyun Tang, Bo Wang, Haijun Wang, Hongliang Wang, Li Wang, Luhao Wang, Sicong Wang, Zhanwen Wang, Zhong Wang, Dong Wei, Jianfeng Wu, Qin Wu, Xuezhong Xing, Jin Yang, Xianghong Yang, Jiangquan Yu, Wenkui Yu, Yuan Yu, Hao Yuan, Qian Zhai, Hao Zhang, Lina Zhang, Meng Zhang, Zhongheng Zhang, Chunguang Zhao, Ruiqiang Zheng, Lei Zhong, Feihu Zhou, Weiguo Zhu

**Affiliations:** ^1^Department of Critical Care Medicine, State Key Laboratory of Complex Severe and Rare Diseases, Peking Union Medical College Hospital, Peking Union Medical College, Chinese Academy of Medical Sciences, Beijing, China; ^2^Department of Surgical Intensive Critical Unit, Beijing Chao-yang Hospital, Capital Medical University, Beijing, China; ^3^Department of Critical Care Medicine, Fujian Provincial Key Laboratory of Critical Care Medicine, Shengli Clinical Medical College of Fujian Medical University, Fujian Provincial Hospital, Fujian Provincial Center for Critical Care Medicine, Fuzhou, Fujian, China; ^4^Department of Critical Care Medicine, Nanjing Drum Tower Hospital, The Affiliated Hospital of Nanjing University Medical School, Nanjing, Jiangsu, China; ^5^Evidence-based Medicine Center, School of Basic Medical Sciences, Lanzhou University, Lanzhou, China; ^6^Department of Critical Care Medicine, West China Hospital of Sichuan University, Chengdu, China; ^7^Department of Critical Care Medicine, The First Medical Center, Chinese PLA General Hospital, Beijing, China; ^8^Department of Intensive Care Unit, The First Hospital of China Medical University, Shenyang, Liaoning, China; ^9^Department of Critical Care Medicine, Beijing Friendship Hospital, Capital Medical University, Beijing, China; ^10^Department of Critical Care Medicine, Northern Jiangsu People’s Hospital; Clinical Medical College, Yangzhou University, Yangzhou, China; ^11^Intensive Care Unit, Sichuan Academy of Medical Sciences & Sichuan Provincial People’s Hospital, School of Medicine of University of Electronic Science and Technology, Chengdu, China; ^12^Department of Critical Care Medicine, Zhongnan Hospital of Wuhan University, Wuhan, Hubei, China; ^13^Department of Critical Care Medicine, The Second Affiliated Hospital of Harbin Medical University, Harbin, Heilongjiang, China; ^14^Department of Information Center, Peking Union Medical College Hospital, Peking Union Medical College, Chinese Academy of Medical Sciences, Beijing, China; ^15^Department of Critical Care Medicine, Chongqing General Hospital, Chongqing, China; ^16^Medical Data Research Institute, Chongqing Medical University, Chongqing, China; ^17^Information Network Center, QiLu Hospital, ShanDong University, Jinan, China; ^18^Department of Computer Science and Engineering, Central South University, Changsha, China; ^19^Department of Information Management, Beijing Jiaotong University, Beijing, China; ^20^Department of Critical Care Medicine, Union Hospital, Tongji Medical College, Huazhong University of Science and Technology, Wuhan, China; ^21^Intensive Care Unit of Cardiovascular Surgery Department, Qilu Hospital of Shandong University, Jinan, China; ^22^National Institute of Healthcare Data Science, Nanjing University, Nanjing, China; ^23^British Chinese Society of Health Informatics, Beijing, China; ^24^Faculty of Automation, Guangdong University of Technology, Guangzhou, China; ^25^Department of Intensive Care Unit, Zhejiang Provincial People’s Hospital, Affiliated People’s Hospital, Emergency and Intensive Care Unit Center, Hangzhou Medical College, Hangzhou, Zhejiang, China; ^26^Department of Intensive Care Unit, National Cancer Center/National Clinical Research Center, Cancer Hospital, Chinese Academy of Medical Sciences and Peking Union Medical College, Beijing, China; ^27^Department of Epidemiology and Biostatistics, Institute of Basic Medical Sciences Chinese Academy of Medical Sciences; School of Basic Medicine Peking Union Medical College, Beijing, China; ^28^Department of Critical Care Medicine, Sun Yat-Sen University First Affiliated Hospital, Guangzhou, China; ^29^Intensive Care Unit, XiangYa Hospital, Central South University, Changsha, China; ^30^National Clinical Research Center for Geriatric Disorders, Xiang Ya Hospital, Central South University, Changsha, China; ^31^Hunan Provincial Clinical Research Center for Critical Care Medicine, Xiang Ya Hospital, Central South University, Changsha, China; ^32^Department of Emergency Medicine, Key Laboratory of Precision Medicine in Diagnosis and Monitoring Research of Zhejiang Province, Sir Run Run Shaw Hospital, Zhejiang University School of Medicine, Hangzhou, China; ^33^Department of General Medicine, Peking Union Medical College Hospital, Peking Union Medical College, Chinese Academy of Medical Sciences, Beijing, China

**Keywords:** machine learning, intensive care medicine, big data, critical care medicine, consensus

## Abstract

The development of intensive care medicine is inseparable from the diversified monitoring data. Intensive care medicine has been closely integrated with data since its birth. Critical care research requires an integrative approach that embraces the complexity of critical illness and the computational technology and algorithms that can make it possible. Considering the need of standardization of application of big data in intensive care, Intensive Care Medicine Branch of China Health Information and Health Care Big Data Society, Standard Committee has convened expert group, secretary group and the external audit expert group to formulate Chinese Experts’ Consensus on the Application of Intensive Care Big Data (2022). This consensus makes 29 recommendations on the following five parts: Concept of intensive care big data, Important scientific issues, Standards and principles of database, Methodology in solving big data problems, Clinical application and safety consideration of intensive care big data. The consensus group believes this consensus is the starting step of application big data in the field of intensive care. More explorations and big data based retrospective research should be carried out in order to enhance safety and reliability of big data based models of critical care field.

## Introduction

The development of intensive care medicine is inseparable from the diversified monitoring data, which specifically presents the clinical manifestations of patients with critical symptoms. These data illustrate a certain clinical phenomena, and represents the nature of disease behind the phenomenon. Intensive care medicine has been closely integrated with data since its birth. The complexity of critical illness makes the traditional reductionist approach to medical research insufficient (1). Critical care research requires an integrative approach that embraces the complexity of critical illness and the computational technology and algorithms that can make it possible (2). Hence, the organic combination of artificial intelligence and critically ill patient data can provide significant assistance for clinical diagnosis and treatment (3). Pirracchio et al. summarize the current application of machine learning for predictive analytics and decision support in the ICU and propose online learning in the future (4). Sanchez-Pinto et al. review the definitions, types of algorithms, applications, challenges, and future of Big Data and data science in critical care (5). There are no concenus of application of big data on the field of intensive care in China so far. Specifically, the conception, clinical research site, standard of dataset, methodology and limitation are not fully exhibited. In this experts consensus, we would like to summarize the problem above and give recommendations based on evidence.

## Consensus formation

This consensus is initiated and formulated by Intensive Care Medicine Branch of China Health Information and Health Care Big Data Society, Standard Committee, and is methodically supported by the Health Data Sciences and Research Institute of Lanzhou University/Research Innovation Unit of Evidence-based Evaluation and Guidelines of Chinese Academy of Medical Sciences/Guidelines for Implementation and Knowledge Transformation Cooperation Center of the World Health Organization. This consensus has been registered on the International Practice Guide Registration Platform (Practice guideline registration for transparency, PREPARE^1^) with the registration number being PREPARE-2022CN566.

**Figure 1:**
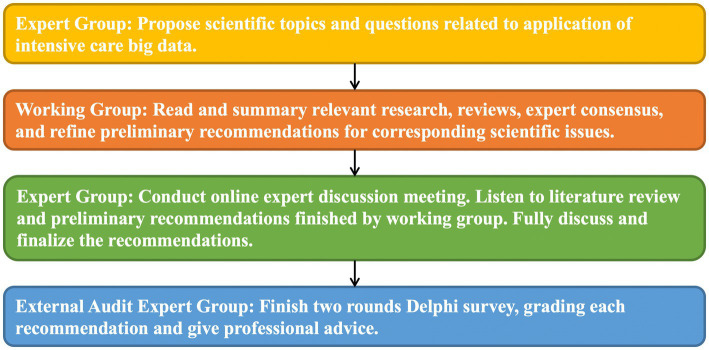
Work flow of formation of consensus.

The consensus development group consists of the consensus expert group, secretary group, working group and external audit expert group. The work flow of formation of consensus are shown in Figure 1. The enrollment criteria and obligation of these groups are shown in Supplementary material S1.

For each recommendation, external audit expert used the Likert scale (Score range: 1–6) to evaluate recommendation degree. Specifically, 6 points for total agree, 5 points for general agreement, 4 points for uncertainty, 3 points for a little disagree, 2 points for disagree and 1 point for total disagree. For each recommendation, if more than 70% external audit expert grade no less than 6 points, the consensus is reached. In this formula, 31 recommendations were put forward. Except for the two recommendations on missing value and outlier value, the remaining 29 recommendations were finalized. The degree of expert recommendation was marked with “Consensus Degree,” which equal to the total number of experts no less than 6 points/total number of experts×100%.

## Consensus text

### The concept, significance and necessity of intensive care data

**Recommendation 1** (5–8)**: The concept of intensive care big data (97% consensus)**

Intensive care big data refers to the datasets with logical connotations formulated by various indicators which are large-scale, multi-heterogeneous, variably dynamic, high-speed and real-time acquisition, low-value density and difficult to analyze traditionally in the whole process of diagnosis and treatment of patients or potential ones with critical symptoms.

**Recommendation 2: The intensive care big data is multi-modal, massive, dynamic, continuous, and objective, and its correct acquisition can provide auxiliary evidential support for diagnosis of critical illnesses and early warning. (98% consensus)**

Background and Evidence:

The monitoring methods used in the intensive care unit are abundant, and the data obtained by the combined use of multiple monitoring equipment have a multimodal characteristic (9–11). According to the needs, multi-parameter sampling can be performed at different levels and time to obtain a large amount of continuous data. Therefore, the intensive care data has the characteristics of abundance (9), dynamics, continuation, and accuracy (5, 12). Correct and effective data processing has a guiding and early-warning role in the diagnosis and treatment of critical illnesses (8). Recently, Epimed Monitor System^®^, a cloud-based ICU management system that includes data of more than 2.5 million hospitalization in the ICU of Brazil, has been deployed to predict the duration of ICU stays, provide guidance for risk assessment of patients becoming long-term ones in the ICU, and help to plan the use of hospital beds (13). Komorowski et al. (14) used reinforced learning techniques to guide patients with sepsis to use fluid or vasoactive medication, and external validation showed that the model made better choices for treatment than intensive care physicians. In the aspect of building predictive models by using data mining techniques, Nemati et al. (15) demonstrated that “AI sepsis experts” can be used for real-time data processing to predict new sepsis within 4–12 h. Although big data research has shown broad prospects for application, at this stage, the number of random clinical trials is small, and various technical models need to be testing prospectively in the clinic to prove their effectiveness and safety (15). In view of the characteristics of individualized and differential conditions in patients with critical symptoms (1), at this stage, the intensive care big data cannot provide maturely clinical guidance and can be used as an auxiliary support tool.

**Recommendation 3: The establishment of a large database for intensive care in China should follow the principles of multiple center, multiple disease and automatic capture, and provide reliable and accurate data support for the application of big data and the development of artificial intelligence. (92% consensus)**

Background and Evidence:

The establishment of a large database for intensive care in China is in the preliminary exploration. By drawing on the experience of existing databases at home and abroad and summarizing the deficiencies of the existing databases, the database can provide a basis for clinical decision-making in China, precise medicine implementation and formulation of medical policies in China. At present, a number of large databases for intensive care have been established abroad, such as the latest version of the Intensive Care Information Database (Medical Information Mart for Intensive Care-IV, MIMIC-IV) (16), the eICU Collaborative Research Database (eICU-CRD) (17), high time resolution ICU dataset (HiRID) (18) and Amsterdam University Medical Centers Database (Amsterdam University Medical Centers Database, AmsterdamUMCdb) (19), etc., mainly based on European and American races. The volume of data is large and the types of data are abundant, but the vital signs are regularly monitored, which are not fully automatically captured, and the scoring system for critical illnesses does not have functions of automatic data collection and integration (20), there is a general lack of software embedding of preliminary data analysis online. The large database for intensive care abroad needs improving in terms of real-time data and availability. Based on the MIMIC database, the researchers conducted in-depth mining of big data to build clinical models. With the help of artificial intelligence and machine learning, artificial intelligence physicians can be used to assist clinical decision-making and provide personalized, clinical and optimal treatment for patients with critical symptoms and improve the prognosis (14).

In recent years, China has been exploring large databases for intensive care, and has successively established a database of ICU infected patients (21), a pediatric intensive care database (PIC) (22), and HeartFailure database (23), etc. The existing large databases for intensive care started late, and their development is not yet mature. They are all single-center databases, with a single type of disease or population. They are limited to the initial collection of early data, and they do not have functions of automatic data capture and data analysis, and the overall quality of data is relatively low and The utilization efficiency is not high. It has not been integrated into clinically artificial intelligence and application technology of big data (24).

Table 1 shows the brief information comparison of major foreign intensive care databases. It can be seen that the existing databases at home and abroad are mainly single-center, and various illnesses may develop into critical one and require admission to ICU for treatment, so it is significant to improve the comprehensiveness of the data. Therefore, the existing single-center or multi-center databases established for certain diseases obviously cannot meet the needs of the vast majority of ICU patients. As we all know, the most widely used database such as MIMIC-III database records vital signs every hour, but for patients with critical symptoms who need continuous dynamic monitoring, this temporal resolution ratio is far from satisfactory. HiRID has a higher temporal resolution ratio than other published datasets, and data storage processes every 2 min (18), which is not yet possible for other databases. To sum up, the establishment of a large database for intensive care in China should follow the principles of multiple centers, multiple diseases and automatic capture to provide data support for the development and application of artificial intelligence.

**Table 1:** Brief information comparison of major foreign databases of critical illnesses.

	MIMIC-III	MIMIC-IV	eICU	HiRID	Amsterdam UMCdb
Sources of included population	Single Center, Large Sample, Beth Israel Deaconess Medical Center in MIT		Multi-center, mainly small and medium-sized hospitals, organized by non-intensive specialists, with patients in 335 ICUs in the United States	Single-center, ICU patients at the University Hospital of Bern, Switzerland	Multi-center, with 20,109 ICU patients in Europe
Country/Region	USA	USA	USA	Switzerland	Europe
Time	2001–2012	2008~2019	2014~2015	2008.1~2016.6	2013~2016
Number of patients	46,520	383,220	139,367	36,098	20,109

**Recommendation 4: Build a large database of patients with critical symptoms in China for their condition monitoring, the research and development of clinical drug and clinical trials can provide the standardized and individualized treatment for patients with critical symptoms. (97% consensus)**

Background and Evidence:

Understanding the relationship between intensive care big data and critical clinic is crucial. The relationship between intensive care big data and the clinic is that: data integration can provide clinicians with manageable, interpretable, operational and treatment plan data, give certain reference to clinical treatment. Data management can provide better personalized and accurate medical guarantee through predicted and prognostic model, It can also use supervised and unsupervised learning algorithm to provide clinical researchers with handy, highly-credible and highly-utilizable database, provide scientific data support for drug development and exploration process, and finally promote the development of intensive care medicine. At present, the application of intensive care big data in clinical practice is gradually increasing, but it is mainly limited to mechanical data collection and manual data processing. The expert group believes that machine learning modeling and multi-disciplinary combination can be used to warn, track and summarize different clinical problems, so as to summarize past experience, warn current decisions and predict future progress.

The first is the application of intensive care big data in clinical decision-making. An RCT study conducted in two community hospitals in 2010 pointed out that remote data algorithms could effectively improve the medical quality of patients with critically symptoms (25). Meanwhile, a review in 2015 showed strategies for the application of big data in the use of antibiotics in patients with critically symptoms. They proposed the concept of AutoKinetics to provide decision support for clinical dosing. And through direct interaction with electronic medical records, they broadened the way to use big data and provided the right dose for each patient at the right time (26). Kindle et al. (27) and Carra et al. (8) summarized the developmental results of all remote algorithms and concluded that machine learning algorithms have important implications for sepsis detection, sepsis management, mechanical ventilation, reduction of false alarms, and prognosis in ICU. In addition, intensive care big data is also of great significance for decision making of clinical care. In 2022, the Stanford medical team developed an unsupervised process mining algorithm to evaluate the quality of care. The final result of the patient cohort had an average compliance score of 0.36. The highest was 0.64, and the lowest was 0.20. The results demonstrated the reliability of big data algorithms for data mining of electronic medical records, and the scheme could also be used to evaluate the quality of care in other diseases (28). In 2022, Jens Michael Boss et al. (29) proposed “ICU Cockpit,” an integration platform of algorithmic model, which pointed out the early warning effect of severe big data on clinical decision-making. Since 2016, the platform has processed over 89 billion data points (979 patients) from 200 signals and laboratory in the analysis, and an infrastructure-based framework has been proposed for deploying and validating intensive care algorithms. It allows algorithms to seamlessly integrate into real-time data streams to generate clinical decision support and predictions in clinical practice (29). The second is the guidance of intensive care big data for clinical research. Taglang and Jackson (30) and Xu et al. (21) expounded the importance of big data to explore clinical trials systematically and, respectively. In the exploration of big data in the past 2 years, a number of studies have carried out analysis of individualized computational models constructed through big data, pointing out risk factors for high mortality in patients with critical symptoms (31–33). Finally, in terms of the relationship between clinical drug R&D and big data, we have not seen any evidence that relevant big data is used in drug R&D in the field of critical care medicine. However, due to the considerable progress of application in drug R&D and big data during recent years (34), we recommend that big data can also be combined with drug development in intensive care medicine. Therefore, the expert group recommends intensive care big data be used to detect changes in clinical practice, but more databases and algorithms and large-scale RCT experiments are needed to jointly promote the development of this field, which is also the future path of clinical practice. We point out that the multidisciplinary and interactive development of intensive care big data can build a large database of critical diseases in China, and ultimately guide the standardized treatment of patients with critical symptoms.

### Clinical scientific issues concerned by intensive care big data in clinical research

**Recommendation 5: It is recommended to use machine learning method to build modeling to make early warning of sepsis, acute kidney injury (AKI), and acute respiratory distress syndrome (ARDS). (94% consensus)**

Background and Evidence:

Research on early warning models for sepsis, AKI, and ARDS is increasing, and most models can provide early warning with good sensitivity and specificity. The ability of different models to predict and popularize needs to be further verified. The expert group believes that machine learning method modeling can be used in the early prediction of the risk of sepsis, AKI and ARDS in ICU patients, so as to reduce the possibility, improve early coping ability, and possibly improve prognosis.

The sepsis early warning model compared with manual screening and scoring, made early and accurate predictions, and achieved external validation. A meta-analysis of sepsis prediction models in 2020 showed that a single machine learning model can be an accurately early prediction of sepsis (AUROC 0.68–0.99) and could replace traditional scores, but heterogeneity between studies limited the evaluation of results (35). A study in 2022 (36) developed a sepsis screening tool by using a learning approach to gradient-boosted supervision that was more sensitive (84.6% vs. 80.4%) and more accurate (28.8% vs. 11.4%) than traditional scoring. A controlled study in 2021 (37) developed an algorithm that accurately predicted sepsis 12 h in advance (AUC 0.94, sensitivity 0.87, specificity 0.87). A multi-center study in 2021 (38) showed the use of a transfer-learning algorithm to enable the validity of the external validation datasets in sepsis.

Early warning models for AKI patients with critical symptoms can make early and accurate predictions, but few models have external validation, clinical interpretability, and high predictive performance in one (39). Studies have shown (40) that the early warning model of AKI has an AUC of 88%, which can predict AKI 6 h in advance. A multi-center study in 2020 showed that the AKI early warning model could predict AKI 48 h in advance, and performed well in both internal and external validation (AUC of 0.86 and 0.85, 0.86 respectively) (41). A 2020 study (42) established a continuous prediction model based on the data of electronic medical record, which could predict AKI in real time during hospitalization, and its performance was significantly better than the one-time prediction model (AUC of 0.724 vs. 0.653).

The ARDS early warning model can make early prediction of ARDS efficiently, and some models can achieve external validation, and some incorporate variables of iconography. A study in 2020 (43) using the XGBoost gradient boosting tree model could accurately predict ARDS 48 h in advance (AUROC of 79.0%). A study in 2020 (44) performed a secondary analysis of prospective study data using the text of radiology reports to build a model that performed well and achieved external validation (C-statistic, 0.78; 95% CI, 0.72–0.84). The diagnosis of ARDS is strongly dependent on iconography, which is, however, not necessarily available at the time of diagnosis or there is uncertainty in its interpretation. This information is called privileged information and uncertainty labels, but the model incorporating variables of iconography is closer to clinical practice. A study in 2021 (44) used a transfer-learning algorithm based on radiographs to build a predictive model that performed well and had external validation (AUROC of 92 and 88%). A study in 2021 (45) successfully used privileged information and a learning model with uncertainty labels to predict ARDS (AUC of 85.78 and 87.01%).

**Recommendation 6: The prediction model based on machine learning can effectively predict the risk of patients at high risk of potential organ damage in the ICU. (89% consensus)**

Background and Evidence:

The proposed early warning scoring system enables medical staff to better identify potential patients with critical symptoms and achieve the purpose of early identification and intervention to improve patient prognosis. However, this scoring system may fail to identify patients until significant deterioration occurs. A systematic review in 2019 (46) found that the early warning score using statistical modeling was more accurate in identifying high-risk patients than weighted early warning (mean AUC of 0.80 vs. 0.73), with one true finding of positive case being 4.9 and 7.1 alarm events required. A similar 2021 systematic review (47) also showed that an early warning system for clinical deterioration based on machine learning could more accurately predict the risk of patient with lower survival rate in the ICU, with an area under the model ranging from 0.57 to 0.97.

Specifically, in addition to the progression of the primary disease, patients with critical symptoms may develop a variety of life-threatening comorbidities. The common ones include failure of circulatory function. In 2020, a study Hyland et al. (18) independently established an early warning system for circulatory failure, which could identify patients at risk of circulatory failure more than 2 h in advance, and successfully conducted external validation in an independent patient cohort. There was also a study by Broch Porcar et al. (48) and they considered that by using data mining, modeling, machine learning and other techniques to generate predictions, risk quantification methods could be developed to predict QTc interval prolongation. The QTc interval risk score showed good predictive performance, with good sensitivity (74% high risk, 67% intermediate risk), specificity (77% high risk, 88% intermediate risk), positive (79% high risk, 55% intermediate) and predictive value of being negative (high risk 76%, intermediate risk 88%). In addition to circulatory function and ECG function, water and electrolyte disturbances are also risk factors for patients with critical symptoms. The Spanish researchers Broch Porcar et al. (48) developed a Spanish national algorithm by reviewing the management of hyponatremia in ICU patients to improve the standardized diagnosis and treatment of hyponatremia. There was also a study (49) that the analysis group of machine learning and the analysis library of collaborative data which were based on the intensive care information system were used to know the area under the curve could be greater than 0.80 when gastrointestinal bleeding in patients was after 5 h, and it had good predictability. In addition to bleeding risk, ICU patients are also at risk of embolism. Deep vein thrombosis (DVT) is associated with high morbidity, mortality, and increased healthcare costs. Researchers (50) developed gradient boosting machine learning algorithms to predict the risk of DVT in patients 12 and 24 h before onset. The area under the curve for the diagnosis of in-hospital DVT obtained by machine learning predictors was 0.83 and 0.85, respectively.

**Recommendation 7: It is recommended to use machine learning method to build modeling to conduct early screening of hospitalized patients, so as to provide help for clinicians intervene early and reduce the severity of the disease. (88% consensus)**

Background and Evidence:

Compared with ordinary patients, patients with critical symptoms often undergo longer hospitalization time, more expense, and poorer prognosis. Early detection of the change of patients’ condition and timely intervention are of significance for preventing the progression of the disease. Machine learning methods can facilitate early screening of diseases and timely treatment of diseases. However, for different subjects, attention should be paid to the correction of heterogeneity before the model is applied, otherwise it will easily lead to wrong clinical guidance. Experts suggest using machine learning method to building modeling for early screening of patients with critical symptoms, so as to help clinicians intervene early and reduce the severity of the disease.

A study published in 2020 evaluated several machine learning methods by using 5-fold cross validation, and applied the XGBoost algorithm to make a AI prediction model for sepsis. The validation results showed that its accuracy = 82% ± 1%; sensitivity = 65% ± 5%; specificity = 88% ± 2%; area under the receiver operating characteristic curve (AUROC) was approximately 0.89, significantly better than the SOFA score (AUROC = 0.596), which might help clinicians deploy appropriate therapeutic regimen, so early and precise responses to this AI algorithm will reduce costs, improve outcomes, and benefit healthcare systems, medical staff, and patients (51). For example, a multi-center and real-world data study in 2020 confirmed that after applying the early warning model in the clinical setting, the in-hospital mortality rate of patients with sepsis decreased by an average of 39.5%, the length of hospital stay decreased by 32.3%, and the 30-day readmission rate of sepsis-related hospitalization decreased by 22.7% (52). In addition to sepsis, machine learning methods have also been used in early screening of other critical illnesses, and a study published in 2021 used a model built with four machine learning methods (Random Forest, XGBoost, GLM-Boost, and LASSO-GLM) to predict pediatric multiple organ dysfunction (MOD). The results showed that the early prediction model of all methods achieved an AUROC of 0.91, and early prediction through risk-based patient monitoring could provide more than 22 h of lead time for the occurrence of MOD, which would play an important role in improving the prognosis of patients (53). However, there were also articles that suggest that clinicians should first calibrate the model according to the heterogeneity of patients before applying the relevant model, so as to avoid misjudgment that might affect clinical decision-making (35). However, in clinical work, first-line clinical staff should pay more attention to the existing scoring system and supervise the actual application, otherwise it will be futile to simply improve the performance of the model without improving the clinical application and response speed (54).

**[Diagnosis]**

**Recommendation 8: It is recommended that the image data of patients with critical symptoms be included in the intensive care database to provide more comprehensive, accurate and timely diagnostic information, so as to guide clinical decision-making through relevant algorithms. (92% consensus)**

Background and Evidence:

There have long been studies using AI in the screening and diagnosis of tumors and the images of infectious foci, and have confirmed its advantages in rapidly processing a large amount of image data, moved the diagnostic “gateway” forward, and avoided missed diagnosis and misdiagnosis (55, 56). The disease state and imaging manifestations of patients with critical symptoms are more complex and diverse, and the optimal timing and scenarios for using artificial intelligence for imaging diagnosis need to be more verified. The expert group believes that AI-assisted imaging diagnosis of ICU patients has good application prospects, and recommends devoting to relevant exploration to improve the efficiency and accuracy of diagnosis and provide reference for clinical decision-making.

A study of 3,078 chest radiographs from 500 ICU patients at Michigan Hospital used directional measurements and deep machine learning features to model ARDS with an accuracy of 83% and an AUC value of 0.79 (57). Cerebellar model arithmetic computer analyzed the supine chest radiograph: the AUC values for the diagnosis of pneumonia and pleural effusion were 0.737 and 0.740, respectively, which were similar to those of imaging experts (AUC values are 0.779 and 0.698) (58). In the outbreak of COVID-19, AI-assisted imaging diagnosis has performed well. Various machine learning methods could not only quickly identify the CT images of COVID-19 (AUC values were between 0.951 and 0.980) from a large number of lung CT images, but also It could predict severe transformation in patients (AUC value was 0.848) (59). The machine learning method combining classical imaging processing and deep learning analyzed CT images of 110 patients with severe subdural hematoma, and showed that the sample recall rate and precision rate were 78.61 and 76.12%, respectively, and the specificity judged based on the severity of the hematoma volume was 92.31%, which could help physicians save decision-making time (60).

In addition to radiological imaging, AI has also been applied in other ICU bedside imaging diagnosis. One study in 2019 showed that the neural network model could detect bedside lung ultrasound B-lines with a sensitivity and specificity of 0.871 and 0.930 (61); two studies in 2021 showed that the neural network model used ultrasound images to diagnose patients with Sepsis early and the accuracy and sensitivity of developing AKI are higher than those of professional radiologists (62, 63). Electrical impedance tomography (EIT) can only roughly show the distribution of ventilation and blood flow in various regions of the lung, but it cannot be quantified as a bedside monitoring index. The neural network model trained by deep learning can calculate information such as lung volume, air flow rate, normalized airway pressure and even transpulmonary pressure from the EIT signal, and AI can also optimize the output image of EIT and even reconstruct the chest image (64).

**Recommendation 9: It is recommended to divide patients with sepsis, acute kidney injury, and acute adult respiratory distress syndrome into phenotypes with different clinical outcomes and treatment responses by means of cluster analysis, and identify patients who are most likely to benefit from specific treatment strategies. (91% consensus)**

Background and Evidence:

Cluster analysis can identify relatively homogeneous groups within heterogeneous populations. Some treatments are only effective in certain groups of people. Clustering techniques were used to classify patients with critical symptoms into distinct phenotypes by significant differences in comorbidities, laboratory indicators, vital signs, clinical outcomes, and treatment responsiveness, identifying groups that benefit from specific therapies. At present, the identification of phenotypes has made research progress in sepsis, AKI, and ARDS, but the accuracy and generalizability of phenotypes still need further verification. The expert group recommends that patients with critically symptoms be divided into different phenotype by cluster analysis to identify those most likely to benefit from specific treatment strategies.

Clinical and/or host response data and machine learning (e.g., latent class analysis and K-means clustering) were used to segment critically-ill patients with sepsis, AKI, ARDS, etc. into distinct phenotypes (65–68). A RCT study in 2021 identified 4 coagulation-based sepsis phenotypes by K-means clustering and used a machine learning means to determine which phenotype would benefit from rhTM (69); another RCT study by Cluster analysis identified 4 clinical phenotypes of sepsis. These phenotypes differed in demographic characteristics, laboratory abnormalities, patterns of organ dysfunction, and were not homologous to traditional patient groups such as site of infection, pattern of organ dysfunction, or disease severity (70); a latent class analysis of an AKI cohort in 2020 identified two phenotypes of sepsis acute kidney injury with distinct clinical outcomes (71); a prospective observational cohort research through unsupervised consensus clustering and machine learning analyzed expression profiles of the whole blood RNA and identified 4 sepsis endophenotypes (Mars 1–4), of which Mars 1 was significantly associated with 28-day mortality. To facilitate clinical application, the study also extracted accurate classification biomarkers for each phenotype (72). Two different ARDS phenotypes have been identified by the LCA method using data from randomized controlled trials of ARDS. These phenotypes had different clinical outcomes. And different treatment responses to positive end-expiratory pressure strategies (73), fluid therapy (74), and simvastatin (75) have been identified.

**[Treatment]**

**Recommendation 10: In specific clinical scenarios, such as decision making for tracheal intubation and intensive care drug decision, it is recommended to build a decision-making model that can be used for clinical treatment based on machine learning algorithms. (74% consensus)**

Background and Evidence:

The condition of ICU patients is usually difficult and critical. Electronic medical record systems, monitors, ventilators and other instruments and equipment can generate massive amounts of vital information data, which far exceeds the ability of ICU doctors to continuously process and correctly interpret them, and affects the effectiveness of clinical decision-making and responsiveness. Artificial intelligence (AI) models can continuously clear, categorize, classify, calculate, and correlate a large amount of data, and make predictions for patients, thereby assisting clinical decision-making and improving the quality and efficiency of critical care.

Several studies have evaluated the clinical impact of applying artificial intelligence techniques such as machine learning to make treatment decisions. In 2018, Komorowski et al. applied reinforcement learning to the sepsis population, and AI clinicians could optimize fluid and vasoactive drug treatment and reduce the fatality rate (14). In 2019, a study established a model to predict urine output in patients with AKI. Compared with the traditional Logistic regression model, the XGBoost model could better distinguish whether patients had volume responsiveness (76).

AI technology has been tried to be applied to clinical situations such as extubation decision-making and optimization of drug treatment for patients. A 2018 retrospective study used machine learning to identify patients requiring prolonged mechanical ventilation (PMV) and those with high risk of tracheostomy (77). In 2021, Fabregat et al. compared three classification learning methods (Logistic regression, XGBoost, and support vector machines) to predict extubation outcomes, which may potentially reduce extubation failure rates (about 9%) (78). Another study in 2021 established a predictive model for accidental extubation through a machine learning algorithm, in which the random forest algorithm obtained the best AUROC of 0.787 (79).

The application of machine learning to optimize the therapeutic effects of anticoagulation, anti-infection and sedation in patients with critical symptoms is still in the exploratory stage. Chen et al. (80), Su et al. (81), Li et al. (82) compared different machine learning methods to predict the therapeutic effect of anticoagulant drugs (citrate, heparin). The scores are overall better than the other models. A single-center retrospective study in 2022 used machine learning and cluster analysis to provide guidance on antibiotic management in patients with critical symptoms (83). Another study in 2022 based on self-attention and residual structure of convolutional neural network (CNN) had a good predictive effect on anesthesia depth monitoring (84). The examples above illustrate the potential role of AI in guiding critical decisions in patients with critical symptoms. But the vast majority of developed ICU-AI models are still in the testing or prototyping stage, and only a few have actually been evaluated in clinical practice. Van de Sande et al. found no studies suggesting the results of integrating AI models in routine clinical practice (85). Research on AI used to guide clinical decision-making is mostly calculated from retrospective and observational datasets. Therefore, in order to have AI directly guide clinical decision-making, it is necessary to conduct a comprehensive analysis of the suggested sequences or strategies derived from such AI systems with more high-quality and prospective studies to be designed.

**[Prognosis and follow-up]**

**Recommendation 11: It is recommended to use machine learning methods to predict the prognosis of patients with critical symptoms. (85% consensus)**

Background and Evidence:

There are more and more predictive models for mortality in ICU patients. Many data models are better at than disease prediction than clinical scoring systems. The sensitivity and specificity of some predictive models still rely on the assistance of clinical scoring systems. AI models in intensive care medicine are mainly generated by retrospective data, with small sample sizes and low reproducibility of conclusions, which are lack of sufficient external validation or prospective evaluation.

There are various machine learning models and algorithms, such as: support vector machines (SVM), Gradient Boosting Decision Tree (GBDT), Logistic regression (LR), adjacent algorithms (KNN, K-Nearest Neighbor), and Random Forest (RF). Studies have shown that the SVM model is a useful tool for early prediction of patients with a higher risk of death upon admission to the ICU. Compared with the early warning score of the SAPS II score, it was better at predicting 7-day mortality. However, the sensitivity and specificity of the SVM model without SAPS II significantly decreased (86). The prediction performance of the machine learning method and the traditional scoring system was further compared according to different diseases. The results were as follows: (1) Sepsis; The results in 2021 showed that GBDT is more accurate than other models (GBDT, LR, KNN, RF, and SVM) in predicting death in patients with sepsis (87). García-Gallo et al. used an assembly algorithm such as SGB to generate a sepsis model that was more accurate in predicting 1-year mortality than traditional scoring systems such as SAPS II, SOFA or OASIS (88). (2) Intracebral Hemorrhage (ICH); Nie et al. (89) indicated that RF was the best model for predicting mortality in ICH patients treated in the ICU, and all machine learning algorithms used to predict mortality in the ICU showed better results compared to the APACHE-II score. (3) Severe acute pancreatitis (SAP); Halonen et al. (90) established an artificial neural network (ANN) model for predicting the severity of acute severe pancreatitis, and the results were better than the Rason score, Glasgow-imrie, APACHE-II, and SOFA scores. The article by Ding et al. (91) also showed that the ANN model could easily screen patients with high risk of death in the early stages of acute pancreatitis.

Finally, it is important to note that the study by Niven et al. (92) showed that a minority of critical care practices with research published in high-profile journals were evaluated for reproducibility; less than half had reproducible effects. This question highlighted the importance of accurate labeling and precise reporting methods, including data preprocessing and functionalization.

**[Auxiliary decision-making system changes the clinical path]**

**Recommendation 12: A clinical decision support system (CDSS) can be used to improve compliance with guidelines for diagnosis and treatment of patients with critical symptoms and the implementation of clinical pathways. (86% consensus)**

Background and Evidence:

Evidence-based clinical diagnosis and treatment guidelines provide standardized and homogeneous diagnosis and treatment strategies for the treatment of patients with critical symptoms. However, compliance with clinical guidelines is not high in routine ICU care, resulting in an increase in avoidable patient mortality (93, 94). A clinical decision support system (CDSS) is a computer program that helps health care workers make decisions. With the clinical application of CDSS, most studies have shown that the application of CDSS can assist ICU physicians in decision making, improve compliance with diagnosis and treatment guidelines, and improve outcomes of patient. However, there are many types of CDSSs. One CDSS is aimed at a certain disease, and the development cost is high. The CDSS based on big data has been applied to clinical decision-making, but it has not been used to change guideline compliance. Moreover, CDSS needs to be integrated with the patient electronic health record system. Due to the different electronic health record systems adopted by different regions or hospitals, the promotion and application of CDSS in different hospitals are limited. Therefore, the expert group believes that CDSS can be used to improve the compliance with the guidelines for diagnosis and treatment of patients with critical symptoms, but CDSS based on big data is still in the stage of research and development. It is recommended that qualified hospitals take the development and clinical application of CDSS based on big data into consideration to improve compliance with guidelines.

As early as in 2011, CDSS, such as a “flow sheet,” can monitor various parameters of patients in real time at the bedside, screen patients with sepsis early and make a series of mandatory treatment measures according to SSC guidelines. The application of CDSS can significantly improve the compliance with SSC guideline of resuscitation bundle strategy, shorten the duration of antibiotic use (90), and reduce hospital mortality (95). In the clinical implementation of lung protective ventilation with low tidal volume, by using CDSS to guide medical staff to set the ventilator mode and support level, the compliance with lung protective ventilation improved, and the level of tidal volume increased significantly after CDSS was discontinued (96). In a study of delirium management, the duration of delirium episodes was significantly reduced, followed the adoption of the tailored ICU delirium guideline CDSS and the duration of coma was reduced, with the brain function improved (97). In another prospective observational study assessing the compliance with AKI guidelines, the CDSS for AKI was integrated into the intensive care information system in the ICU and found the proportion of patients with worse condition from stage 1 AKI, and the proportion of inappropriate use of enoxaparin dose as well as that of morbidity rate of patients with AKI was significantly reduced (98). It can be seen that CDSS can improve guideline compliance. However, there is currently no big data-based CDSS application in clinical practice to improve guideline compliance, which needs to be confirmed by further research in the future.

### Establishment, standards and principles of a large database for intensive care

**Recommendation 13: It is recommended to build a intensive care medicine database and data analysis platform. (98% consensus)**

Background and Evidence:

The intensive care database can provide a good data foundation and new ideas for clinical medical research, which in turn can improve the understanding of diseases. For example, in Sepsis 1.0 (99), sepsis was defined as a systemic inflammatory response syndrome (SIRS) caused by infection. Although various diagnostic indicators were more complete in Sepsis 2.0 (100), it still continued the standard of Sepsis 1.0. However, the diagnostic criteria of infection and SIRS cannot accurately describe the disease characteristics of patients, such as different primary diseases, different symptoms and mortality of patients. In 2016, Sepsis 3.0, which was mainly based on big data analysis, was born (101), which defined sepsis as life-threatening organ failure caused by the body’s uncontrolled response to infection, i.e., infection and organ function diagnosis. Patterns, making the definition of Sepsis more adaptable to pathophysiology and easier to implement in clinical practice. It can be said that the intensive care medicine databases that have been constructed abroad, such as the Medical Information Mart for Intensive Care (MIMIC) and the eICU Collaborative Research Database (eICU-CRD) (17), are used in clinical practice. The role played in diagnosis and treatment has gradually become prominent. At present, the pace of establishing a intensive care big data platform has also been accelerated in China, but most of them are limited to individual databases in each hospital, and there are still some deficiencies in data exchange and influence. Therefore, we recommend building a intensive care medicine database and data analysis for Chinese people platform to strengthen discipline construction and improve the level of treatment for patients with critical symptoms.

**Recommendation 14: It is recommended to form a standard normative intensive care dataset. (97% consensus)**

Background and Evidence:

Standard and normalized datasets are the basis for big data applications and facilitate data collaboration between research centers in different regions. There is a lot of information obtained by ICU equipment and instruments, and the data can be included in a reasonable and standardized manner and classified, so that they can be used more fully and conveniently. At present, there are many big data information systems for intensive care medicine at home and abroad. These information systems divide clinical data into different data elements according to specific classification standards, and then use specific data collection methods to acquire and analyze data. Referring to basic structure and data standard of the national electronic medical record (102), Beijing local standard - intensive care medicine dataset and the intensive care medicine database widely used in the field of medical research (103), the recommended standard data set should include the following data sets: (1) Basic information data of patients; (2) Diagnostic information data of patients; (3) Monitoring data of Patients; (4) Drug use data of patients; (5) Laboratory information data of patients; (6) In and out data of patients; (7) Imaging data of patients; (8) Etiology data of patients. See Table 2 for details.

**Table 2:** Standard datasets.

Basic information data of patients	Time information on patient admission and discharge, demographic information, source of admission, ICU category, time of death, etc.
Diagnostic information data of patients	All disease diagnosis information during the patient’s stay in the ICU; the main diagnosis needs to be distinguished from the secondary diagnosis
Monitoring data of Patients	Routine vital signs, ventilator parameter information, blood purification parameter information, aortic balloon counter pulsation parameter information, the mental state, the score information, etc.
Treatment data of patients	The route of administration, use time and drug dose of all drugs during the patient’s stay in the ICU; the name, time and related information of the operation; the name, time and related information of the treatment operation, etc.
Laboratory information data of patients	Laboratory examination information during the patient’s stay in the ICU, such as sampling time, specimen type, test items, reference range of normal values, etc.
In and out data of patients	Data of all fluids entering and expelling from the body during the patient’s stay in the ICU, including fluid type, entry and exit route, time, etc.
Imaging data of patients	Text reports related to radiographic imaging during patient stay in the ICU
Etiology data of patients	The etiological data collected during the patient’s stay in the ICU, including sampling time, specimen type, etiological name, etiological drug susceptibility, etc.

It is also recommended that adjustments can be made in combination with actual conditions such as hospital disease conditions, information centers, laboratory testing items and other objective conditions. For example, based on acute respiratory distress syndrome, sepsis, acute kidney injury and other common diseases in intensive care medicine to build a special disease database, which is necessary to strengthen the sampling frequency and categories of intensive care information related to special diseases. For example, the acute respiratory distress syndrome database needs to further collection of biomarkers, etc.; The sepsis database requires further collection of vasoactive drugs, etiology collection, organ function assessment, etc.

**Recommendation 15: It is recommended to select automatic collection for objective data first. For data that cannot be automatically collected for the time being, targeted collection should be carried out in combination with research needs, data sources and data types. (92% consensus)**

Background and Evidence:

The data collection process should follow the principles of comprehensiveness, multi-dimensionality, efficiency and timeliness. In view of the many data sources and rich data structures in the ICU, it is recommended to use automated data collection technology to realize the data collection process so as to avoid human errors affecting the use of subsequent data.

Data in the ICU can be broadly classified into “phenotypic data” and “physiologic data.” Phenotypic data include demographics, age, sex, laboratory values, and physician and nursing records. Phenotypic data collection can be queried and extracted from electronic medical records (EMRs). Relevant content can be obtained through Python or API, and the required attribute content can be extracted from it. Physiological data include vital signs (blood pressure, heart rate, respiratory rate, core temperature) and other parameters (intracranial pressure, EEG) generated by bedside monitoring equipment. If the data interface of the device can be obtained through various software manufacturers, data collection and aggregation can be realized through the interface docking method. If some devices cannot obtain the data interface, collecting all the data generated by the target device can be tried to acquire the underlying data exchange of the system, the network package between the client and the database, which can convert the data into with restructure and output to new database, based on underlying IO request and network analysis technologies.

Alarms in the ICU, such as ECG leads, blood pressure cuff detachment from patients, completion of infusion pump or air bubbles in tubing, high airway pressure, air leak, or apnea in mechanical ventilation ventilators, etc., which can be classified into the type of physiological data. This part of the data can be collected by collecting logs from log sources of various devices. Continuous waveform data is more complicated to acquire due to its continuous nature and high sampling rate. In recent years, many studies have used time series databases and unstructured databases such as InfluxDB, MongoDB, etc. to explore the writing, storage, and query processes of various continuous-time signals, which can solve the storage-transmission-exchange-exploitation problem (104). For image data, since most of the images are currently stored in the PACS system, it is necessary to clarify whether to collect from the equipment (CT machine, ultrasound machine, etc.) or through the PACS docking port (105).

**Recommendation 16: It is recommended to optimize standard system for intensive care big data, standardize multi-center source data, and constrain standard codes, measurement units, field standards, as well as naming dictionaries to ensure the homogeneity and standardization of the use of the large database for intensive care. (95% consensus)**

Background and Evidence:

“Information integration, standards first” (106), the construction of large databases for intensive care must be implemented in accordance with the corresponding norms and standards, the standard codes, measurement units, field standards, and naming dictionaries, and it is constrained by standard norms to ensure the subsequent modeling and application process. The consistency of data processing ensures the standardized production of data from the source, and lays the foundation for the construction, data integration, data exchange and data sharing of large databases for intensive care. Intensive care big data are multi-modal data with high privacy and diverse sources, and have the characteristics of multiple data dimensions, good timeliness, high value density and high data quality. The “phenotypic data” and “physiological data” in the ICU can be classified into structured discrete data, time series data, and unstructured text data, image data, and audio-video data (107). The main contents are as follows: (1) Discrete data: basic information and routine data of patients’ physical sign, including a series of discrete data such as gender, age, blood type, height, weight, etc., which are mainly characterized data. These data volumes are small and stable. (2) Time series data: mainly physiological data, including time series data of various vital sign parameters such as blood oxygen, heart rate, and ECG. These data are closely related to the real-time symptoms of patients, with high real-time performance, strong continuity, and large datasets. (3) Image data: mainly physiological data, including a large amount of image data such as ultrasound and radiation. These image data are large in volume and are important reference data for diagnosis and operation. (4) Text data: a large amount of text data about patient medical records and diagnostic results, mainly for representation data, including electronic medical records, surgical records, inspection reports, etc. Among all data types of critical diseases, time series data, image data and text data have high information value density and play an important role in clinical diagnosis, treatment and decision making.

Due to the uneven level of informatization in each center and a wide range of coverage, the above-mentioned data formats for intensive care are complicated and difficult to integrate. After negotiation, multiple centers have formulated unified data fields, contents and formats for the big database for intensive care, and established a standard system. For example, for the standardization of image data, the level of imaging departments in different hospitals varies, and multiple centers need to negotiate the image quality standards for uploading compressed original images. For different types of data, in order to ensure the standardization of large databases for intensive care, data governance rules for different types of data can be formulated, and the system will automatically clean the data when it enters the database, supplemented by manual review if necessary to ensure data quality. For the quality assessment of inbound data, it can be measured from normative (the extent to which the data conforms to data standards, data models, business rules, metadata or authoritative reference data), integrity (the extent to which data elements are assigned values according to data rules), accuracy (the degree to which the data accurately represents the true value of the real entity, “real object” that it describes), consistency (the degree to which the data does not contradict the data used in other specific contexts), timeliness (the degree to which the data is correct over time), and accessibility (the degree to which data can be accessed), which are six aspects to manage and evaluate (108).

**Recommendation 17: It is recommended to establish a data security system to ensure the security of data storage, processing, sharing and use. (98% consensus)**

Background and Evidence:

The information security system in China mainly includes five technical tasks: risk assessment and grade protection, monitoring system, cryptography and network trust system, emergency response system, and disaster preparedness. The security level of information system is divided into five levels, and the levels from one to five are gradually increased. Centering on the “Network Security Law,” “Data Security Law” and “Personal Information Protection Law,” China carries out the construction of data classification system. In terms of data security, the security of the data itself (using modern cryptographic algorithms to actively protect data) and security of data protection (active protection of data using modern information storage methods) must both be paid attention to. New security issues need to be addressed in an environment of big data, including balancing privacy and utility, analyzing and governing encrypted data, and verifying authenticated and anonymous users. With the continuous expansion of the application scope of intensive care big data, the content is becoming richer and more valuable with a large amount of sensitive personal information. A security system and a safety management responsibility system for intensive care big data should be established to ensure the security in data storage, opening, and processing (109, 110).

When storing data, system security reinforcement as well as software and hardware architecture design in a distributed environment (such as Apache Hadoop) should be done well. Strict fine-grained access control and risk registration management strategies should be set for static data, and privacy-related data storage should realize classified isolation data encryption (such as AES, RSA, SHA-256 and other encryption methods) and other security technical means, dynamic data classification and identification of important sensitive data should be through encryption and dynamic audit capabilities, using TLS (transport layer security technology) to communicate between cluster nodes and maintain confidentiality during transmission, and enabling unified management across platforms (endpoints, mobile devices, networks, and storage systems) (106).

During data processing, the software architecture and network configuration should be designed according to the database volume and access method, especially for multi-center, and the appropriate hardware architecture should be designed according to the software architecture. And policy configuration such as network security should be done to ensure data security. After the data is authorized to be processed by other parties, the most important question is whether there is misuse and malicious restoration of sensitive data during the processing, whether it complies with laws and regulations, and whether it complies with the privacy clauses agreed by both parties (106). In multi-party computation, data leakage is avoided through system policy design such as data desensitization (111) and federated learning (112).

When sharing data, measures such as data desensitization, rights management, and log auditing should be taken to ensure data security. Data cannot be unconditionally open to the public or third parties. Consideration should be given to the fact that sensitive information can be easily restored after a single information is desensitized through multi-source collisions which may lead to security risks, therefore, only point-to-point sharing, or multilateral transactions based on certain special constraints, such as sharing health records, patient medication information, medical images and other information about intensive care big data. Whether the data sharing is justified or not should be comprehensively weighed on the occasions of the data and the subject’s right to know.

### Ways and methods to solve big data problems in intensive care medicine

**[Type of data]**

**Recommendation 18: It is recommended to use processing methods of digital signals such as filters to preprocess time series data, deep learning to process image data, use Natural Language Processing (NLP) technology to process unstructured text data. (93% consensus)**

Background and Evidence:

From the perspective of machine model building, intensive care data can be roughly divided into four categories: numerical time series data, numerical non-series data, text data, and image data. Among them, numerical data can be divided into two categories according to the collection density: (1) time series data, or “streaming data,” including electrocardiogram, arterial and intracranial pressure, hemodynamic monitoring, ventilator data, brain waves and other data with relatively high collection frequency; (2) non-sequential data, or “sparse data,” including blood gas analysis, laboratory test results, medical history and other data with relatively low collection frequency. Different types of data can be combined to improve the accuracy of AI prediction models (113), provide decision support under complex and uncertain diagnostic conditions (114), and better adapt to the clinical real-time data environment.

For time series data, before further pattern recognition or other processing through different algorithms, processing methods of digital signals such as filters are usually used for preprocessing. The main purpose is to use various mathematical methods to strip components of different frequencies in the signal for targeted treatment. For example, in electrocardiogram (ECG) data processing, a five-minute moving average is often used for low-pass and high-pass filtering (29, 115, 116), and when building an EEG signal model, Narula et al. also used a band-pass filter to remove baseline drift and high-frequency interference (117).

For non-series data, the processing skills are mainly reflected in solving the problems of data (parameter) outliers and missing values, screening and dimensionality reduction according to different algorithm models. After the corresponding preprocessing of the data, whether it is a simple algorithm such as linear regression and logistic regression, or a sophisticated algorithm such as lifting algorithm and reinforcement learning (14, 118), it can achieve good results in the corresponding scene. So no special recommendation is made.

For image data, such as CT, pathological slices, ultrasound images, etc., most of them are processed by deep learning (such as convolutional neural network CNN, etc.) and other tasks (119–122). In particular, Walsh et al. believed that deep learning methods can directly extract important features from images, which could help to generate novel biomarkers and more accurate image analysis tools (123).

For unstructured text data, such as narrative text in EMR, as well as radiology, pathology reports, etc., the content can be mined and processed through natural language processing technology to obtain pathological information, social environment information, etc., which can be combined with the existing expert knowledge base (such as the unified medical language system, etc.) as a supplement to improve the accuracy of related prediction models, and show a speed and accuracy that exceeds manual processing (124–126). In particular, natural language processing for Chinese, ICTCLAS system, THULAC toolkit, etc. are all good auxiliary tools, but the Sinicization of knowledge bases such as UMLS (or other Chinese medical knowledge bases) needs to be demonstrated in the literature.

**[Data preprocessing]**

**Recommendation 19: It is recommended to use resampling methods to deal with unbalanced datasets. (78% consensus)**

Background and Evidence:

In intensive care medical datasets, unbalanced data is very common. Unbalanced data refers to the uneven distribution of the number of samples among each category in the classification task, and there will be a particularly large gap, which will greatly affect the final performance of the prediction model. For example, a small number of death samples in intensive care medicine datasets carry important information about mortality prediction, but are ignored because the model is insensitive to data imbalances. In response to the phenomenon of data imbalance, the expert group recommends using resampling methods to process imbalanced data, which are mainly divided into three types: undersampling, oversampling and synthetic oversampling. Undersampling is the random sampling of fewer samples from most classes so that the data tends to be balanced. Edited Nearest Neighbors (ENN) is the most typical undersampling method. Oversampling is to generate more labeled samples according to the sample rules with fewer sample labels so that the data tend to be balanced. Synthetic Minority Over-sampling Technique (SMOTE) is an oversampling technique that generates synthetic samples for the minority class. In order to reduce the fitting problem caused by oversampling and undersampling, a method combining oversampling and undersampling is extended to deal with data imbalance on this basis, such as SMOTEENN, SMOTETomek, etc. In the study of using machine learning to predict atrial fibrillation, Tiwari et al. used a variety of sampling methods to deal with the imbalance problem that the data in the control group was much more than that in the experimental group, and compared the data under different sampling methods, and finally chose the random oversampling method according to the classifier effect (127). Papp et al. used SMOTE sampling to synthesize samples from the minority class for the class-imbalance problem and analyzed the synthesized new data results through cross validation and confusion matrix (128).

**Recommendation 20: It is recommended to convert original categorical variables and numerical variables into variables that can be directly processed by machine learning algorithms through one-hot encoding, sequential encoding, etc. (83% consensus)**

Background and Evidence:

The function of variable category transformation is to convert the original category of intensive care medical data containing the above information into a form suitable for data mining and easy for model understanding. The transformation of variable categories makes the original data more tidy and consistent through operations such as encoding. It is recommended to use methods such as one-hot encoding and sequential encoding. One-hot encoding is a common numerical processing method for unordered categorical variables, with “1” to indicate that it belongs to this category, and “0” to indicate that it does not belong to this category. One-hot encoding will add new variables to the original variables. The number of new variables being added is the number of types. Ordinal coding is a common numerical processing method for ordinal categorical variables. This coding makes numerical one according to the different degrees represented by the ordinal variables, such as scores about a patient’s health status from 0 to 5.

**Recommendation 21: It is recommended to use dimensionality reduction methods such as principal component analysis to perform variable screening of high-dimensional features in intensive care datasets. (90% consensus)**

Background and Evidence:

In most research problems of intensive care big data, the datasets used usually have high-dimensional feature variables, which can easily lead to overfitting problems and increase training costs. Therefore, it is necessary to extract important features through variable screening to achieve the purpose of data dimensionality reduction. Experts recommend principal component analysis, variance selection, univariate feature selection, regularization models, feature ranking based on machine learning models, and recursive feature elimination methods.

Principal Component Analysis (PCA) is a popular general feature dimensionality reduction method, which can be used to reduce the dimensionality of various types of data such as numerical values, texts, and images. Essentially, multiple variables are synthesized into a few independent components, and each component can reflect the information of the original variable, which can improve the learning speed and reduce the training cost. Variance selection is a simple feature selection method that filters features by removing features with low variance. Univariate feature selection usually uses statistical test methods such as chi-square test and F test, or measures such as Pearson correlation coefficient and distance correlation coefficient to determine the relationship between variables. The regularization model is mainly divided into L1 regularization and L2 regularization. By adding additional constraints or penalty terms to the loss function of the existing model, it can prevent overfitting and improve the generalization ability of the model. L2 regularization is more stable than L1 regularization and is more favorable for the understanding of features. Regularization models are often used in feature selection of medical data. In the study on early triage of COVID-19 patients with critical symptoms, Liang et al. selected 10 statistically significant variables as predictors by the Lasso method (129). Many machine learning methods can achieve feature scoring, such as feature ranking by measuring feature importance. Therefore, it is recommended to use the selected machine learning model to complete feature selection, including SVM, random forest, decision tree, XGBoost, LGBM and other models. By adjusting the calculation parameters of feature importance, the feature ranking of different methods can be obtained. This method is convenient, effective and easy to understand the relationship between the model and features, but it is needed to verify the model fitting effect by means of cross-validation. In addition, recursive feature elimination methods can be considered to screen the features of intensive care medical data.

**[Model Construction]**

**Recommendation 22: It is recommended to select supervised learning, unsupervised learning and reinforcement learning models for critical disease prediction and identification according to different scenarios and different data types. (97% consensus)**

Background and Evidence:

The intensive care unit monitoring system collects a large number of the patients’ respiratory, hemodynamic, neurological and clinical data, and its electronic medical record system also records the patient’s clinical treatment and medication information in detail. The data types include types of text, digital and image. Through the processing and analysis capabilities of big data by machine learning algorithms, key features of the data can be mined to assist in diagnosis and decision making. Machine learning algorithms can be classified into supervised learning, unsupervised learning, and reinforcement learning, depending on whether the dataset has labels. Among them, supervised learning can learn and summarize models, including decision trees, support vector machines, random forests, naive Bayesian models, artificial neural networks and other models; Unsupervised learning models can discover hidden patterns without manual annotation or data grouping, which can find potential similarities and differences in the data. Common algorithms include k-means, principal component analysis, hierarchical clustering, etc.; Reinforcement learning can learn the best behavior or mode that should be taken from experience. The model type should be selected according to the data type and medical task. Among them, for numerical data and clinical prediction problems, supervised learning models can be used; For text data, natural language processing models and unsupervised learning models can be used; For image data, Convolutional Neural Networks (CNN), Recurrent Neural Network (RNN) can be used for medical image recognition and segmentation; For clinical auxiliary decision-making tasks, reinforcement learning models can be used. According to the literature survey, the usage scenarios of three different learning methods include: (1) Supervised learning: prognosis prediction, phenotype classification, analgesic and sedation strategy selection, mortality risk prediction, disease severity prediction, prediction for length of stay in the ICU, etc. (2) Unsupervised learning: disease pattern mining and representation based on electronic health records (EHR). (3) Reinforcement learning: decision making of treatment plan, recommendation of fluid volume, robot-assisted surgery, etc.

Specifically, examples of the usages and indications for the three types of learning are as follows:

1. Supervised learning: Prognosis prediction and dose recommendation for heparin patients (82); monitoring and adjustment of Local citric acid anticoagulation (80); prediction of in-hospital mortality risk in patients with critical symptoms (124, 130) prediction of mortality risk in patients with candidemia (125), prediction of the severity of lung ultrasound in ICU patients (126), etc.
2. Unsupervised learning: Phenotype classification and sedation strategy selection in mechanically ventilated patients (131); temperature pattern recognition in patients with critical symptoms (67), blood pressure pattern recognition (132); subtype of diseases extracted from electronic health record data (133, 134).
3. Reinforcement learning: Dynamically provide optimal treatment plan and select intravenous fluids and vasopressor doses for patients in the ICU (135).

**Recommendation 23: It is recommended to use a causal inference model to explore and discover causal relationships in the intensive care field. (89% consensus)**

Background and Evidence:

The model system of causal inference is built on the basis of causal-heuristic learning and reasoning. It conducts in-depth mining of relevant data to extract causal structure, and conducts causal-heuristic estimation. It studies the influence of intervention variables on prognosis and obtains the key index of prognosis evaluation. The directions involved include causal discovery, causal structure learning, causal inference, causal deep learning, etc. In response to the need for poor ICU prognosis or poor survival rate, as well as the need to accurately determine the influencing factors of prognosis, it is advisable to use the frameworks of DoWhy, CDT and CausalML and establish a causal-heuristic learning inference and decision-making evaluation system based on the database of specialized diseases and multi-center of intensive care.

First, implement big data-driven causal structure identification, mine causal relationships, and conduct feature analysis, effect analysis, and interpretability analysis. Richens et al. (136) proposed a counterfactual diagnostic strategy for expected failure and expected adequacy, breaking the traditional diagnosis method of diseases based on symptoms and narrowing the scope of possible conditions by using counterfactual questions. Wei et al. (137) described the causal relationship between some variables in the recommended system from the perspective of causal inference and solved the influence of popularity bias on the model from the perspective of counterfactual inference. Goudet et al. (138) used deep learning methods to propose a causal generative neural network (CausalGNN), which exploited conditional independence and distribution asymmetry to discover bivariate and multivariate causal structures, and learned functional causal models from observational data to figure out a causal roadmap between clinicopathological features.

In addition, the causal effect was further estimated on the basis of the causal relationship, and machine learning methods such as generalized random forest (GRF) (139) were used to calculate CATE and HTE to predict the difference in prognosis under different ICU intervention methods and research the degree of impact on prognosis by intervention variables. Tan et al. (140) used an approach like adversarial training to give an interpretable means for recommended systems. The advantages of these methods are that the data can be used to reason about the source characteristics of heterogeneity to estimate a series of estimators, which also apply to high-dimensional data and missing data and have good interpretability. Through techniques based on causal discovery and estimation, learning the most discriminative characterization, discovering diagnostic basis and key characteristic indexes, judging prognosis accurately, and providing effective interventions for clinical treatment can be realized.

**[Verification of the model]**

**Recommendation 24: It is recommended to add external validation to internal validation of the model. (94% consensus)**

Background and Evidence:

Model validation is the process of evaluating the predictive performance of a model after it has been constructed. The importance of model validation is reflected in measuring the prediction accuracy of the prediction model, feeding back the model building process, and adjusting the model building ideas if necessary. The model verification idea is relatively mature at present, and there is a relatively consistent method consensus. In practice, model verification is mainly divided into internal verification and external verification. The expert group believes that the following methods can be used to evaluate the model validation process.

Internal verification: In general, verification based on their own data (internal verification) is required. That is, a part of the data (like 80% of the total) are randomly selected as the training set for building the prediction model, and the rest of the data are used as the test set to evaluate the performance of the model. In order to verify that the model has good performance on newly generated clinical data, “spatio-temporal division” can be added to the random division, which is the data of the latest period specially divided as an independent validation set (141). In order to improve the estimation robustness of the evaluation indicators, K-fold cross-validation can be used (18). Divide the data set into K parts (such as 10 parts), use K-1 data to build a prediction model, use the remaining data for verification, repeat K times, and take the average of the K times of model prediction evaluation indicators as the accuracy index of the final model. The implementation of internal verification is relatively simple, but since the training set and test set are both derived from the same data, the model extrapolation ability (i.e., “generalization” ability) is relatively weak.

External verification: Different regions and hospitals may have differences in data distribution due to differences in population, disease characteristics, and diagnosis and treatment habits. In order to verify that the model has good extrapolation, it is recommended to perform external validation on multi-center data from different regions and different hospitals.

**Recommendation 25: It is recommended to use indicators such as sensitivity, specificity, F1 score, and AUC to evaluate the performance of classification models, and indexes such as R** ^**2**^ **, MSE, RMSE, and MAE to evaluate the performance of regression models. (91% consensus)**

Background and Evidence:

During model validation, a series of evaluation metrics should be used to measure model performance (i.e., predictive effect). For classification model and regression model, different indicators are used for evaluation.

Performance evaluation indicators of classification model: For classification models (models whose predicted values are categorical variables), sensitivity (also known as recall), specificity, F1 score, precision, AUC (Area Under Curve) and other metrics to evaluate the performance are generally used (3). Among them, the F1 score is the harmonic value of sensitivity and positive accuracy rate, and the larger the value, the better the model performance is. AUC is the area under the ROC curve drawn by “1-specificity” and “sensitivity.” The larger the value, the better the model performance is. When the sample categories are not balanced, it is recommended to use the area under the PR curve, AUPRC, to evaluate model performance.

Performance evaluation indicators of regression model: For regression models (models whose predicted values are continuous variables), R^2^ (R squared, coefficient of determination, coefficient of determination), Mean Squared Error (MSE), Root Mean Squared Error (RMSE), Mean Absolute Error (MAE), and other indicators are generally used to evaluate the performance. The closer the determinant coefficient, R^2^, is to 1, the better the model performance is. The closer MSE, RMSE and MAE are to 0, the better the model performance is.

**[Model interpretability]**

**Recommendation 26: It is recommended to explore the interpretability of the model to facilitate the clinical transformation of complex machine learning models. The recommended model interpretation methods include Feature Importance, LIME, and Shapley. (91% consensus)**

Background and Evidence:

AI models based on big data training in intensive care medicine are often complex, and their complexity is mainly reflected in the large number of parameters and the complex functional relationship between various parameters. Such complex models are often not conducive to clinicians to analyze the pathophysiological mechanisms, and it is difficult to determine the causal relationship between variables, which seriously hinders the clinical transformation of AI research results. The interpretability of the model is considered as an effective way to solve the above problems. Understanding characteristics, classification, and prediction of indicators, and then understanding why a machine learning model makes such a decision, and what features play the most important role in the decision allow us to judge whether the model is in line with common sense. For example, an AI doctor trained by a reinforcement learning model is used to treat septic shock (14). The AI prompts the need to increase norepinephrine while appropriately limiting fluid replacement. Understanding the mechanism behind such an algorithm is critical for the reliability of the model. If the algorithm tells you that you need to increase the dose of norepinephrine for the patient because their main contradiction is peripheral vasodilation, rather than fluid deficiency, it can greatly enhance the confidence of the physician in the use of this model, because the diagnosis and treatment made by AI decision-making is consistent with clinical pathophysiological changes.

In addition, several other methods are also used for model interpretability exploration (142). Feature importance can be used. Its main working principle is to change the arrangement of the data in a certain column of the data table and keep the rest of the features unchanged to see how much it affects the prediction accuracy.

Locally Interpretable Agnostic Modeling (LIME) is an algorithm (143) that provides a novel technique to interpret the results of any predictive model in an interpretable and trustworthy way. It works by training an interpretable model locally around the predictions it wants to explain. In layman’s terms, select a sample and a point near the sample, and then train a simple model to fit. Although the simple model cannot be effective on the complete data set, it is at least effective near this point. The characteristics of this simple model are human-analyzable, and the trained weights can also represent feature importance.

The Shapley value was proposed by Loyd Shapley, a professor at the University of California, Los Angeles, USA, to solve the problem of contribution and profit distribution in cooperative games. In cooperation of N persons, the contribution of individual member is different, and the distribution of income should also be different. The ideal distribution method is: contribution = income; Is there a quantifiable method for the distribution of contribution and income? The Shapley method is one such method, where the Shapley value of a feature is the average marginal contribution of that feature across all feature sequences.

### Clinical application of intensive care big data

**Recommendation 27: It is recommended to transform and promote early warning tools that meet critical needs. (91% consensus)**

Background and Evidence:

The construction of early warning tools can make early predictions for the risk of various adverse events in the ICU, thus helping clinicians to take timely measures to prevent problems before they occur, reduce the incidence of adverse events in patients effectively, and improve early response capabilities. At present, although early warning models have been constructed and verified for the occurrence and prognosis of a variety of critical diseases at home and abroad, there is still insufficient research to truly conduct large-scale clinical trials to evaluate their application value. A Big-data clinical trial (BCT) of an early warning tool was implemented in terms of injury and disease deterioration. However, there are still differences in the predictive performance of different early warning tools in different application scenarios, and further promotion and verification are needed. So far, no mature disease-targeted early warning tools have been launched at home and abroad. The expert group believes that it is possible to use AI technology to provide early warning for various adverse events in the ICU. At the same time, it is necessary to carry out BCT research to further verify the clinical practical value of early warning tools, so as to achieve early detection, diagnosis and treatment of diseases.

For the early warning of sepsis, Shimabukuro et al. (144) conducted a BCT study in 2017 and found that patients who used the early warning tool for sepsis shortened the length of hospital stay significantly (10.3 days vs. 13.0 days, *p* = 0.042), and the in-hospital mortality rate was reduced significantly (8.96% vs. 21.3%, *p* = 0.018). However, a single-center BCT study conducted by Semler et al. (145) found that the application of a sepsis electronic warning system neither improved the completion of the 6-h bundle of sepsis (*p* = 0.159), nor improved clinical outcomes (including ICU fatality rate, days in ICU, days of vasoactive drug use).

For the early warning of acute kidney injury (AKI), a large multi-center BCT study in the United States in 2021 (146) found that the AKI early warning system did not improve disease progression in patients (*p* = 0.67). However, BCT evidence from the United Kingdom (147) and China (148) found that although the AKI early warning system could not improve the mortality rate of patients, it could significantly improve the early identification rate of AKI (RR: 1.12, 95% CI: 1.03–1.22, *p* < 0.01) and AKI diagnosis rate (7.9% vs. 2.7%, *p* = 0.001). Another BCT study from the United States found that the AKI electronic automatic alert system did not improve the composite outcome (maximum creatinine change, the need for dialysis or death) within 7 days of patients (*p* = 0.88) (149).

For the early warning of disease deterioration, the Escobar et al. (150) conducted a multi-center BCT study in 2020 that included a total of 43,949 people (15,487 people in the intervention group and 28,462 people in the control group). And it found that early warning tool for disease progression can significantly reduce patient mortality rate (adjusted RR: 0.84, 95% CI: 0.78–0.90, *p* < 0.001).

**Recommendation 28: It is recommended to use the information system for intensive care as a carrier to access real-time data and output recommendations for decision making. (91% consensus)**

Background and Evidence:

The condition of patients with critical symptoms is complex and fast-changing, and ICU equipment and instruments have a large amount of information, so the data dimension and the update frequency is high. The application carrier should be effectively integrated with the hospital information system, which can obtain high-dimensional information in real time, and be equipped with a prediction model. Based on Hadoop distributed processing technology, Xia et al. (33) designed a big data analysis system for intensive care medicine, and conducted a performance test through the “Study on the Effect of Xuebijing on AKI-related Sepsis” (33). The information system of intensive care big data can integrate ICU high-dimensional information, obtain analysis data in real time, and use it as a carrier for results of intensive care big data such as prediction models and scores (151). Boss et al. (29) developed an online real-time ICU decision support platform that could be used to collect multimodal waveform data and AI-based computational disease modeling, calling it “ICU Cockpit.” In the cohort of 979 patients admitted to this 12-bed neurocritical care unit since 2016, the total number of data points processed and stored by the “ICU Cockpit” platform has been approximately 88.9 billion (29). Based on the intensive care information system, Zhang Suzhen et al. used the XGBoost model to integrate relevant parameters and performed machine learning to predict the risk of AKI in patients with septic shock. The sensitivity of the prediction results was 73.3%, the specificity was 71.7%, and the accuracy was 72.5%. Compared with the traditional score, it was significantly improved (152).

When there is no information system of intensive care medicine, the intensive care big data can also be equipped with a online prediction tool of web page, APP, applet, or bedside form and other carriers. Flechet et al. developed a prediction model for acute kidney injury, AKI predictor, and conducted a multicenter prospective cohort study to verify the prediction effect of clinicians and AKI Predictor. The performance of the two at ICU admission was: AUROC was 0.80 [0.69–0.92] and 0.75 [0.62–0.88] (*n* = 120, *p* = 0.25), the net benefit ranges were 0–26% and 0–74%. The machine learning-based AKI predictor achieved similar discriminative performance to physicians in predicting AKI-2 and AKI-3, with a higher overall net benefit because physicians overestimated the risk of AKI. This showed that AKI Predictor has added value to the doctor’s prediction. The study also came with an online version of the predictive model^2^ (153).

With the development of Internet of Things technology, 5G technology, database technology, etc., the carrier to realize the application of intensive care big data in the future should focus more on the “dynamic holographic prediction system” that obtains ICU information in a comprehensive and real-time way, analyzes the data and makes real-time predictions.

**Recommendation 29: It is suggested that the current practice of intensive care diagnosis and treatment should still be led by clinicians with the use of big data technology to coordinate to improve medical efficiency and ensure medical quality and safety. (98% consensus)**

Background and Evidence:

In recent years, the development of applications of intensive care big data has made rapid progress, and a large number of articles have been published, including prediction of diseases, early warning of risks, and real-time guidance of clinical medication. In the foreseeable future, big data applications can assist ICU clinical diagnosis and treatment activities. However, at the same time, applications of big data still have problems such as lack of clinical integration, lack of high-quality verification, poor interpretability, few application scenarios, and ethics. Therefore, this consensus believes that because of the current developmental level of big data applications, it is advisable to be guided by existing evidence and clinical experience to assist the diagnosis and treatment, and improve the quality and efficiency of medical care with the help of big data technology.

Big data models produce seemingly accurate results through complex computations, but often fail to provide end users with the logic behind them (154). AI is weak in determining causality, at least its interpretability does not meet current clinical needs. Models developed based on intensive care big data are often more accurate in predictions when validating data from the same population, but the results may be unreliable when tested in external populations (155). In clinical practice, the diagnosis and treatment process is often highly subjective, especially for patients with critically complex symptoms, and their plans for diagnosis and treatment also have large individual heterogeneity, resulting in low reliability of the ICU model (156). In summary, most of the current research is still in the exploited phase and lacks effective external validation (157). Therefore, unnecessary interventions or changes in treatment strategies that are not supported by scientific evidence may lead to medical safety issues such as overmedication or treatment failure.

When these algorithms are developed into intelligent assistance systems deployed as alerting tools, they must be concise and accurate enough to prevent alert fatigue and thus avoid delays in clinical decision-making (158, 159). Considering the scientific preciseness, the maturity and stability of AI-driven models are less convincing for clinical practice to a certain extent, and indiscriminate development and use of data models may lead to overdiagnosis and waste of resources. In addition, the clinical application of intensive care big data also faces ethical issues. At present, the hidden dangers of big data applications in terms of patient privacy and safety responsibility cannot be ignored. First of all, the establishment of the database will inevitably involve data of patient privacy, and protecting patient privacy has become a problem that must be solved in the development of intensive care big data. It is not appropriate to develop a medical database at full speed without guaranteeing privacy and security. Secondly, in terms of application security, in the process of big data-assisted clinical diagnosis and treatment practice, if a medical safety accident occurs, computer algorithms cannot be responsible for clinical decision-making with the current developmental level of ethics and AI. In order to avoid mistakes and abuses in the big data system for diagnosis and treatment, the clinician must act as the person in charge of clinical decision-making to “be responsible for” big data applications.

## Discussion

With the increase of computing power and data scale, the emergence of large models has enabled AI systems to handle more complex and massive tasks, improving the model’s performance and generalization ability, which also brings new opportunities for critical big data applications. As a “double-edged sword,” the application of big data science in intensive care has pros and cons. This consensus reach a consensus on five parts: conception, important scientific issues, standards and principles of database, methodology in solving big data problems, clinical application and safety consideration of intensive care big data. All recommendations has been summarized in Table 3. Actually, this is the starting step of application big data in the field of intensive care. In order to ensure data security and ensure the professionalism of the model, the medical industry needs a medical vertical domain big language model based on professional mapping knowledge domain and high-quality data. More explorations and big data based retrospective research should be carried out in order to enhance safety and reliability of big data based models of critical care field.

**Table 3:** Summary of recommendations on the application of intensive care big data.

Part	No.	Recommendation contents	Consensus degree
1. The concept, significance and necessity of intensive care data	1	The concept of intensive care big data: Intensive care big data refers to the datasets with logical connotations formulated by various indicators which are large-scale, multi-heterogeneous, variably dynamic, high-speed and real-time acquisition, low-value density and difficult to analyze traditionally in the whole process of diagnosis and treatment of patients or potential ones with critical symptoms	97
	2	The intensive care big data is multi-modal, massive, dynamic, continuous, and objective, and its correct acquisition can provide auxiliary evidential support for diagnosis of critical illnesses and early warning	98
	3	The establishment of a large database for intensive care in China should follow the principles of multiple center, multiple disease and automatic capture, and provide reliable and accurate data support for the application of big data and the development of artificial intelligence	92
	4	Building a large database of patients with critical symptoms in China for their condition monitoring, the research and development of clinical drug and clinical trials can provide the standardized and individualized treatment for patients with critical symptoms	97
2. Clinical scientific issues concerned by intensive care big data in clinical research	5	It is recommended to use machine learning method to build modeling to make early warning of sepsis, acute kidney injury (AKI), and acute respiratory distress syndrome (ARDS)	94
	6	The prediction model based on machine learning can effectively predict the risk of patients at high risk of potential organ damage in the ICU	89
	7	It is recommended to use machine learning method to build modeling to conduct early screening of hospitalized patients, so as to provide help for clinicians intervene early and reduce the severity of the disease	88
	8	It is recommended that the image data of patients with critical symptoms be included in the intensive care database to provide more comprehensive, accurate and timely diagnostic information, so as to guide clinical decision-making through relevant algorithms	92
	9	It is recommended to divide patients with sepsis, acute kidney injury, and acute adult respiratory distress syndrome into phenotypes with different clinical outcomes and treatment responses by means of cluster analysis, and identify patients who are most likely to benefit from specific treatment strategies	91
	10	In specific clinical scenarios, such as decision making for tracheal intubation and intensive care drug decision, it is recommended to build a decision-making model that can be used for clinical treatment based on machine learning algorithms	74
	11	It is recommended to use machine learning methods to predict the prognosis of patients with critical symptoms	85
	12	A clinical decision support system (CDSS) can be used to improve compliance with guidelines for diagnosis and treatment of patients with critical symptoms and the implementation of clinical pathways	86
3. Establishment, standards and principles of a large database for intensive care	13	It is recommended to build a intensive care medicine database and data analysis platform	98
	14	It is recommended to form a standard normative intensive care dataset	97
	15	It is recommended to select automatic collection for objective data first. For data that cannot be automatically collected for the time being, targeted collection should be carried out in combination with research needs, data sources and data types	92
	16	It is recommended to establish a standard system for intensive care big data, standardize multi-center source data, and constrain standard codes, measurement units, field standards, as well as naming dictionaries to ensure the homogeneity and standardization of the use of the large database for intensive care	95
	17	It is recommended to establish a data security system to ensure the security of data storage, processing, sharing and use	98
4. Ways and methods to solve big data problems in intensive care medicine	18	It is recommended to use processing methods of digital signals such as filters to preprocess time series data, deep learning to process image data, use Natural Language Processing (NLP) technology to process unstructured text data	93
	19	It is recommended to use resampling methods to deal with unbalanced datasets	78
	20	It is recommended to convert original categorical variables and numerical variables into variables that can be directly processed by machine learning algorithms through one-hot encoding, sequential encoding, etc.	83
	21	It is recommended to use dimensionality reduction methods such as principal component analysis to perform variable screening of high-dimensional features in intensive care datasets	90
	22	It is recommended to select supervised learning, unsupervised learning and reinforcement learning models for critical disease prediction and identification according to different scenarios and different data types	97
	23	It is recommended to use a causal inference model to explore and discover causal relationships in the intensive care field	89
	24	It is recommended to add external validation to internal validation of the model	94
	25	It is recommended to use indicators such as sensitivity, specificity, F1 score, and AUC to evaluate the performance of classification models, and indexes such as R^2^, MSE, RMSE, and MAE to evaluate the performance of regression models	91
	26	It is recommended to explore the interpretability of the model to facilitate the clinical transformation of complex machine learning models. The recommended model interpretation methods include Feature Importance, LIME, and Shapley	91
5. Clinical application of intensive care big data	27	It is recommended to transform and promote early warning tools that meet critical needs	91
	28	It is recommended to use the information system for intensive care as a carrier to access real-time data and output recommendations for decision making	91
	29	It is suggested that the current practice of intensive care diagnosis and treatment should still be led by clinicians with the use of big data technology to coordinate to improve medical efficiency and ensure medical quality and safety	98

## Author contributions

LS and SL: write and translate the Chinese version of this consensus and overall planning of the discussion and finalize the draft. YLo: organize and supervise the writing of this article. CC, KC, MC, YCheng, YCui, QD, TG, XG, HH, JH, CH, RH, HJ, JJ, YLa, JuL, LuL, JiL, XL, ZM, HM, YuS, QS, WS, ZT, HaW, LuW, SW, ZhaW, ZhoW, DW, QW, JYu, YY, HY, HZ, MZ, CZ, RZ, and LeZ: literature review and write the origin version of the Chinese version of this consensus. YChen, RD, MD, BH, XH, LiL, WL, YLi, FL, XiaS, XiuS, YoS, YiS, BW, HoW, LiW, JW, XX, JYa, XY, WY, QZ, LiZ, ZZ, FZ, and WZ: supervise and provide consulting of manuscript. All authors contributed to the article and approved the submitted version.

## Footnotes

^1^http: //www.guidelines-registry.org

^2^ https://www.akipredictor.com/en/

## Glossary

AES: Advanced encryption standard
AI: Artificial intelligence
AKI: Acute kidney injury
ANN: Artificial neural network
APACHE-II: Acute physiology and chronic health evaluation-II
API: Application programming interface
ARDS: Acute respiratory distress syndrome
AUC: Area under curve
AUPRC: Area under precision recall curve
AUROC: Area under receiver operating characteristic curve
BCT: Big-data clinical trial
CATE: Conditional average treatment effect
CDSS: Clinical decision support system
CNN: Convolutional neural network
COVID-19: Corona virus disease 2019
CT: Computed tomography
DVT: Deep vein thrombosis
ECG: Electrocardiograph
EEG: Electroencephalogram
EHR: Electronic health records
eICU-CRD: eICU collaborative research database
EIT: Electrical impedance tomography
EMR: Electronic medical records
ENN: Edited nearest neighbors
GBDT: Gradient boosting decision tree
GRF: Generalized random forest
ICH: Intracerebral hemorrhage
ICTCLAS: Institute of Computing Technology, Chinese Lexical Analysis System
ICU: Intensive care unit
KNN: K-nearest neighbor
LASSO-GLM: Least absolute shrinkage and selection operator-generalized linear models
LGBM: Light gradient boosting machine
LIME: Locally interpretable agnostic modeling
LR: Logistic regression
MAE: Mean absolute error
MIMIC: Medical information mart for intensive care
MOD: Multiple organ dysfunction
MSE: Mean squared error
NLP: Natural language processing
PACS: Picture archiving and communication systems
PCA: Principal component analysis
PIC: Pediatric intensive care
PMV: Prolonged mechanical ventilation
RCT: Randomized controlled trial
RF: Random forest
RMSE: Root mean squared error
RNA: Ribonucleic acid
RNN: Recurrent neural network
RR: Risk ratio
RSA: Ron Rivest, Adi Shamir and Leonard Adleman Algorithm
SAP: Severe acute pancreatitis
SAPS: Simplified acute physiology scores
SIRS: Systemic inflammatory response syndrome
SMOTE: Synthetic minority over-sampling technique
SOFA: Sequential organ failure assessment
SSC: Surviving sepsis campaign
SVM: Support vector machine
THE: Heterogenous treatment effects
THULAC: Thu lexical analyzer for Chinese
TLS: Transport layer security technology
UMLS: Unified medical language system
USA: United States of America
