# Supplementary material for: Chinese experts’ consensus on the application of intensive care big data
Source: Front Med (Lausanne). 2024 Jan 8;10:1174429. doi: 10.3389/fmed.2023.1174429 (PMC10804886; doi:10.3389/fmed.2023.1174429)
Supplement: Supplementary file 1 [file Table_1.docx]

**Supplement material 1**

| **Group Name** | **Criteria** | **Obligation** |
| --- | --- | --- |
| **Expert group**  **(n=29)** | - Has rich experience in the application of intensive care big data. - Has representative of regions and disciplines, covering intensive care medicine, information science, mathematics, tutorial methodology, etc. | - Propose scientific topics and raise questions related to application of intensive care big data. - Listen to literature review and preliminary recommendations finished by working group. - Take part in discussion in online expert meeting and finalize the recommendations. |
| **Working group**  **(n=41)** | - Has certain preliminary practice of the test of intensive care big data. Their main responsibilities are to relevant literature and make preliminary recommendations. | - Read evidence from guidelines, consensus, systematic evaluation, meta-analysis, evidence of RCT, search information from the PubMed database, and institute / association website documents related to intensive care big data (time range: from establishment of the database to June 2022) - integrate documents from the consensus expert group and wrote preliminary recommendations and evidence. |
| **Secretary Group**  **(n=2)** | - Has knowledge of critical care medicine and big data. - Has coordination and organizational skills | - Fully responsible for the coordination and management of the consensus. - integrated the consensus on different issues, and organize symposium of each section. |
| **External Audit Expert Group**  **(n=69)** | - Has membership of Intensive Care Medicine Branch of China Health Information and Health Care Big Data Society, Standard Committee and Chinese Writing Panel’s Consensus on the Application of Intensive Care Big Data | - Finish Two rounds of Delphi surveys and provide written opinion of the consensus. |
